# Single-dose liposomal amphotericin B (AmBisome^®^) for the treatment of Visceral Leishmaniasis in East Africa: study protocol for a randomized controlled trial

**DOI:** 10.1186/1745-6215-12-66

**Published:** 2011-03-06

**Authors:** Tansy Edwards, Raymond Omollo, Eltahir AG Khalil, Sisay Yifru, Brima Musa, Ahmed Musa, Monique Wasunna, Peter G Smith, Catherine Royce, Sally Ellis, Manica Balasegaram, Asrat Hailu

**Affiliations:** 1 MRC Tropical Epidemiology Group, London School of Hygiene & Tropical Medicine, UK; 2 Drugs for Neglected Diseases initiative Africa, Centre for Clinical Research, Kenya Medical Research Institute, Kenya; 3 Institute of Endemic Diseases, University of Khartoum, Sudan; 4 Gondar University Hospital, Gondar, Ethiopia; 5 Kenya Medical Research Institute, Kenya; 6 Drugs for Neglected Diseases initiative, Geneva, Switzerland; 7 Faculty of Medicine, Addis Ababa University, Addis Ababa, Ethiopia

## Abstract

**Background:**

AmBisome^® ^is an efficacious, safe anti-leishmanial treatment. There is growing interest in its use, either as a single dose or in combination treatments. In East Africa, the minimum optimal single-dosage has not been identified.

**Methods/Design:**

An open-label, 2-arm, non-inferiority, multi-centre randomised controlled trial is being conducted to determine the optimal single-dose treatment with AmBisome^®^.

Patients in the single-dose arm will receive one infusion on day 1, at a dose depending on body weight. For the first group of patients entered to the trial, the dose will be 7.5 mg/kg, but if this dose is found to be ineffective then in subsequent patient series the dose will be escalated progressively to 10, 12.5 and 15 mg/kg. Patients in the reference arm will receive a multi-dose regimen of AmBisome^® ^(3 mg/kg/day on days 1-5, 14 and 21: total dose 21 mg/kg). Patients will be hospitalised for approximately one month after the start of treatment and then followed up at three and six months. The primary endpoint is the status of patients six months after treatment. A secondary endpoint is assessment at day 30. Treatment success is determined as the absence of parasites on microscopy samples taken from bone marrow, lymph node or splenic aspirates. Interim analyses to assess the comparative efficacy of the single dose are planned after recruitment of 20 and 40 patients per arm. The final non-inferiority analysis will include 120 patients per arm, to determine if the single-dose efficacy 6 months after treatment is not more than 10% inferior to the multi-dose.

**Discussion:**

An effective, safe single-dose treatment would reduce hospitalization and treatment costs. Results will inform the design of combination treatment studies.

**Trial Registration:**

ClinicalTrials.gov NCT00832208

## Background

Visceral Leishmaniasis (VL) is a parasitic disease that is transmitted by phlebotomine sandflies, and is fatal if not treated. Estimates suggest there are 500,000 cases per year, with 90% of cases occurring in India, Bangladesh, Nepal, Sudan, Kenya, Ethiopia and Brazil [1,2]. In East Africa, the disease has been known to occur in cyclical epidemics, one of which resulted in over 100,000 deaths in Southern Sudan between 1984-94 [3,4]. Parasitological diagnosis is determined by lymph node, bone marrow and spleen aspirates. The mainstay of control in East Africa, where the disease is believed to be largely anthroponotic, has been case finding and treatment. Treatment options for VL in the region has been limited to antimonials (sodium stibogluconate or glucantime), given intramuscularly (IM) or intravenously (IV) for 28-30 days. This long and relatively toxic treatment is inconvenient for patient management. Two other treatments; paromomycin, a 3- week IM treatment, and miltefosine, a 4-week oral treatment, have recently been developed and registered for VL in India. Both are in development in Africa, but both require relatively long courses, have some toxicity and there are contraindications and issues with compliance [2].

AmBisome^®^, a liposomal formulation of Amphotericin B has been shown to be safe and effective against leishmaniasis. In trials in India, an efficacy of 90% was obtained with a single dose of 5 mg/kg [5,6]. A major limitation of this treatment has been its cost but recently, the drug has been offered at a preferential price for the treatment of VL by its producer, Gilead. This has raised considerable interest in its use either as a single dose treatment or as part of a combination treatment where a single dose of AmBisome^® ^might be followed by a short course of a companion drug such as miltefosine[7]. There are limited data on the use of AmBisome^® ^in East Africa. One small phase II trial in Kenya showed an efficacy of 20% at a total dose of 6 mg/kg, 90% at a total dose of 10 mg/kg and 100% at a total dose of 14 mg/kg [8].

At the time of developing this study protocol, there was a recommendation made to use AmBisome^® ^in East Africa at a total dose of 21 mg/kg [9] but this recommendation was based on experience with the drug in India and it is not known if this is the appropriate dose for use in the treatment of African patients. That there might be geographical difference in drug efficacy is illustrated by a recent study conducted by the authors, as members of the Leishmaniasis East Africa Platform (LEAP), and Drugs for Neglected Diseases initiative (DNDi), which showed that the efficacy of paromomycin for VL, is lower in East Africa, than in India[10]. There is a clear need 1) to demonstrate the efficacy of AmBisome^® ^given under the standard recommended multiple dose regimen; and 2) to determine if lower single doses of AmBisome^® ^could be used to treat VL.

## Methods/Design

### Study Design

An open-label, non-inferiority, multi-centre, individually randomised controlled trial.

### Main Hypothesis

The hypothesis is that a sufficiently large single dose of AmBisome^® ^is safe and is not inferior in efficacy to a multiple dose regimen of AmBisome^®^.

### Main Objective

To determine the minimum single-dose treatment regimen of AmBisome^® ^that is efficacious and safe.

### Setting

Trial sites are in three locations, each in endemic areas for VL. These are Gondar University Hospital in Amhara State - Ethiopia; Arba Minch Hospital, Southern Nations, Nationalities and Peoples State - Ethiopia; and Kassab Hospital, Gedarif State, Sudan. All data collected in the trial are analysed in the data centre located at the Coordination Centre for the trial at the Kenya Medical Research Institute (KEMRI) in Nairobi, Kenya.

### Outcome measures

#### Primary outcome

*Definitive Cure *- defined as absence of parasites 6 months after the end of the 30 hospitalisation period following the initiation of treatment (i.e., 7 months after the start of treatment), confirmed by splenic or bone marrow aspiration.

#### Secondary outcomes

*Initial Cure* - defined as parasitological clearance, measured on day 30 after initiation of treatment on day 1, confirmed by lymph node, splenic or bone marrow aspiration.

### Sample Size

The final analysis will be based on the primary efficacy endpoint of definitive cure. Assuming a true efficacy of 95% in both arms, 120 patients per arm will provide 80% power to detect if the efficacy of the single-dose treatment regimen is no more than 10% inferior to the reference multi-dose regimen[11].

**Figure 1:**
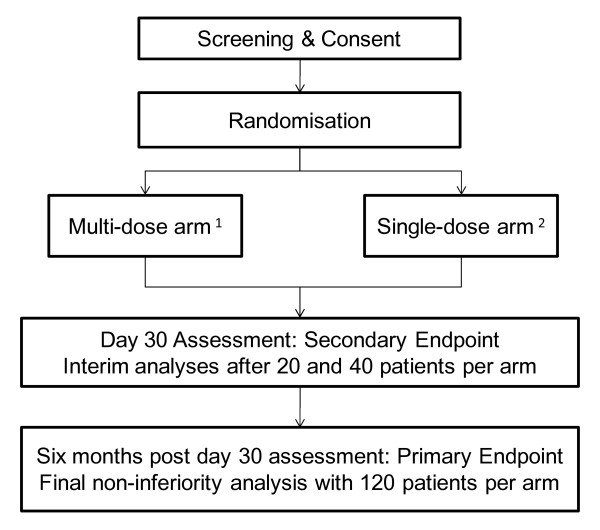
**Trial Flow CONSORT Diagram**. ^1 ^dosage: 3 mg/kg on days 1-5, 7 and 14 (total dose 21 mg/kg). ^2 ^possible single-doses of 7.5, 10, 12.5 & 15 mg/kg on day 1.

The trial design allows for a dose escalation in the single-dose arm at the pre- specified interim analysis time points if the single-dose regimen is found to be performing poorly, compared to the multi-dose and based on a test of difference in the proportion of patients achieving initial cure, measured at the secondary efficacy endpoint on day 30 (Figure 1). Based on a two-sided significance test for a difference in proportions at the 5% level assuming 95% efficacy in the reference arm, 20 patients per arm will provide approximately 80% power to detect a difference if the test arm is less than 60% effective and 40 patients per arm will provide 80% power to detect a difference in efficacy if the test arm is less than 70% effective [12].

### Choice of NI margin

A margin of 10% below an assumed efficacy of 95% was considered acceptable based on clinical acceptability given the limited availability of possible treatments for VL and sample size considerations based not only on power but on recruitment time projections.

### Allocation

Patients will be randomised to one of two treatment arms; either the reference multi- dose regimen or the test single-dose regimen.

Block randomisation by site will be used to minimise imbalance in multi-centre interim analyses. The user-written program *ralloc *for Stata will be used to obtain the final sequence[13]. The process of randomisation generation and the resulting final randomisation list, produced by the trial statistician will be kept securely at the DNDi Data Centre. Sequentially numbered, sealed, opaque envelopes will conceal random allocation to treatment until immediately prior to receipt of first treatment administration.

Blinding of the patients or clinicians was not considered feasible or ethical; placebo injections in the single-dose arm would have caused additional unnecessary discomfort to patients.

### Interventions

#### Multiple-dose regimen

A total dose of 21 mg/kg given as a 3 mg/kg intra-vascular (IV) infusion on days 1,2,3,4,5,14 and 21, was chosen as the reference treatment regimen, expected to achieve efficacy of 95% [9,14].

#### Single-dose regimen(s)

The trial will compare only one single-dose test regimen to the multi-dose regimen during any one recruitment period but the trial design does allow for testing of incrementally higher single-doses in later recruitment periods if lower doses are ineffective (see above and Type of Analysis section).

Based on experience from treatment trials for VL in India and Kenya[15], either 7.5 mg/kg or 10 mg/kg is likely to be the lowest dose to show suitable efficacy in a single-dose regimen [14-16] although the efficacy of treatment regimens used in India can be substantially lower in African setting [8,10]. The trial will begin with a 7.5 mg/kg single-dose regimen in the test arm. If this regimen is found to be ineffective according to pre-specified criteria at the time of an interim analysis, the trial design allows for recruitment and randomisation into the two arms to continue with a higher single-dose (see Type of Analysis section). Higher single doses to be used, if 7.5 mg/kg is ineffective, are 10 mg/kg,12.5 mg/kg and 15 mg/kg. It is considered that 15 mg is the maximum dose that can be safely administered as a single-dose [15].

### Screening

#### Inclusion criteria

Aged 4 years or older; VL proven by parasitological examination of splenic aspirate, lymph node or bone marrow aspirate; haemoglobin >4 g/dL; fever for more than 2 weeks; written informed consent to participate (for children, by parent or guardian); HIV negative status.

#### Exclusion criteria

'In extremis' signs/symptoms of severe VL; receipt of anti-leishmanial treatment or investigational (unlicensed) drugs within previous 6 months; known underlying chronic disease, such as severe cardiac, pulmonary, renal, or hepatic impairment; renal function tests (serum creatinine) outside the normal range; liver function tests more than 3 times the normal range; platelet count less than 40,000/mm^3^; known alcohol abuse; pregnancy or lactation; concomitant acute drug usage for malaria and bacterial infection, pneumonia within previous 7 days; known hypersensitivity to AmBisome^® ^or amphotericin B.

In addition, patients presenting with severe dehydration should be re-hydrated before consideration for trial entry and those presenting with acute bacterial co-infection e.g. malaria, pneumonia must have these infections treated prior to trial entry. In the Sudanese site, recruitment until the end of the first interim analysis period will include those aged 12 years and above before widening to age 4 years and above, at the request of the Sudanese Ethics Committee.

### HIV-status and VCT

All patients (or parents/guardians) will be offered Voluntary Counselling & Testing (VCT) for HIV screening/testing in accordance with national guidelines. This trial specifically requires HIV negative patients, therefore patients who refuse counselling and testing, or who test positive will be excluded from the trial, but will be treated for VL according to national guidelines and if HIV positive referred to national HIV treatment programs for assessment and treatment with anti-retrovirals (ARVs) if indicated.

### Consent

Standardised consent forms, adapted to the local context, translated into local languages and approved by ethics committees are to be used. Signatures or thumbprints will be obtained for consent, with witnesses in the case of illiterate subjects. Parents or guardians will be asked to provide consent for all children and in Ethiopia, depending on their age and level of comprehension, children will be asked to give their assent, as required by local regulations.

Patients who do not meet inclusion criteria or who do not give consent will be treated outside the trial, according to national treatment guidelines.

### Type of analysis

The final analysis will be based on a comparison of definitive cure of the single-dose treatment regimen to the reference treatment regimen. If the treatments in the two arms are equally efficacious, the trial is powered such that the confidence interval around the difference in efficacies of the two regimens will be no more than 10% (non inferiority margin Δ = 0.1). The primary analysis will be by intention to treat (ITT). A per-protocol (PP) analysis will exclude patients deemed to have major protocol deviations but include minor deviations, where major and minor deviations are pre-specified prior to recruitment.

Two interim analyses are planned after recruitment of a pre-specified number of patients per arm, to allow for rapid elimination of inadequate dosage regimens. The interim analysis will be assessed at the secondary endpoint, initial cure, measured on day 30, where treatment is initiated on day 1 (Figure 1).

The first single-dose regimen tested will be 7.5 mg/kg body weight. Interim analyses are planned after recruitment of 20 and 40 patients per arm. Randomisation will pause once the desired number of patients for each interim analysis has been recruited, in order to carry out the interim analysis and assess if the trial will continue with the current single-dose or a higher single-dose. If interim results indicate a Fisher's test of a difference with a p-value ≤ 0.05, randomisation will re-start with the next highest dose in the single-dose regimen arm. Interim analyses will take place as pre-specified for each dosage tested in the single-dose treatment arm.

### Schedule of Assessments and Expected Side Effects

During the month that patients are hospitalised, formal weekly assessment will take place to monitor vital signs and other clinical characteristics (Table 1). During these weekly assessments and during follow up, safety will be assessed by means of haematological, urinalysis and biochemical monitoring (Table 1). Known side effects include infusion related fever and shivers [6,17]. Vital signs will be measured during infusions and patients will be asked if they experience discomfort during the infusion. In addition, patients will be asked daily during treatment and at each visit during follow up if they have suffered any side-effects or adverse events (AE). Electrocardiograms (ECG) will be performed where clinically indicated by patient's signs or symptoms. Site investigators will use their judgment to determine the degree of certainty with which each adverse event is attributed to drug treatment. Causality will be classified as; not related, unlikely, suspected (reasonable possibility) or probable. The assessment and classification of intensity/severity and causality in relation to VL and the study treatment will be based on the investigator's clinical judgment and pre-specified criteria according to common terminology criteria (CTC) [18] and applicable in the study population. Serious adverse events (SAE) will be recorded as such if the event is fatal or considered life threatening, disabling or incapacitating or results in re/hospitalisation, prolonged hospital stay or is associated with congenital abnormality, cancer or overdose (either accidental or intentional), in addition to any experience suggestive of a significant hazard, contra-indication, side effect or precaution that might be associated with the use of the drug. All SAEs will be reported to and reviewed by the Medical Coordinator within 24 hours and reported to the Data and Safety Monitoring Board (DSMB) within 7 days. Patients with SAEs or AEs will be followed-up until the condition resolves or stabilises.

**Table 1:** Data Collection and Assessment Schedule

Assessments	In-patient assessment (day)							Follow-up (months)	
	
	BL	1	2,3,4,5	7	14	21	30	3	6
Clinical assessment (BP, body temperature, weight, height^1^, spleen size)	X			X	X	X	X	X	X

Haematology (HB, WBC, Platelet)	X			X	X	X	X	X	X

Biochemistry^2 ^(Urea, creatinine, ALT, AST, Na^+^, K^+^, Mg^2+^)	X		X	X	X	X	X	X	X

CARPA	X	X		X	X				

Pharmacodynamic Assessment	X		X	X	X		X	X	X

Urinalysis (blood, protein, glucose)	X		X	X	X	X	X		

Parasitology (splenic, LN, BM aspirates)^3^	X						X	X	X

HIV test	X								

Electrocardiogram Examination^4^	X			X	X	X	X		X

Pregnancy test	X								

Dosing - including monitoring of vital signs^5^		X	X		X	X			

Adverse events^6^	X	X	X	X	X	X	X	X	X

BL = Baseline, BP = blood pressure, HB = Haemoglobin, WBC = white blood cell count, AST = aspartate aminotransferase, ALT = Alanine transaminase, Na^+^= Sodium, K^+ ^= Potassium, Mg^2+ ^= Magnesium, CARPA = complement activation related pseudo allergy products, LN = lymph node, BM = bone marrow, HIV = Human immunodeficiency virus

^1 ^Body height measurements on Day 0 only

^2 ^Day 3 only

^3 ^Parasitological investigations to be undertaken in the event of clinically significant abnormalities at 3 months follow-up

^4 ^ECG if clinically indicated

^5 ^Single-dose arm monitoring on day 1 only

^6 ^Treatment emergent adverse events have onset at anytime between day 1 and day 60 for each patient

### Rescue medication

In the event of failure to respond to treatment, clinical deterioration or relapse at any time during the study, rescue treatment will be given for patients in the single-dose arm as a full course of AmBisome^®^. In the multi-dose arm and in the single-dose arm for patients withdrawn from treatment due to an adverse reaction, patients will be treated with sodium stibogluconate (SSG) at 20 mg/kg/day for 30 days by intramuscular injection, and intravenously if indicated.

### Concomitant Medication

Details of all concomitant medication taken during the study will be recorded in the case report form (CRF) with indication, daily dose, route and dates of administration.

### Dissemination of Results

An interim analysis report presenting the efficacy (95% confidence intervals) in each arm and the p-value from a Fisher's test of a difference will be provided to the DSMB. This report will also contain a listing of serious adverse events. The DSMB will review the results and report back to the coordination group, to confirm if the stopping rule for a single-dose has been met. Adverse event listings are to be provided at the request of the DSMB. The DSMB, through the Chair may request additional efficacy and safety data if they have concerns relating to trial conduct or other ethical and safety issues [19]. At the end of the trial, the analysis report will be circulated to Principal Investigators, DSMB, Ethics Committees, Ministries of Health and Drug Regulatory Authorities.

### Ethical Approval

Ethics approval has been obtained from national and local Ethics Committees in Sudan and Ethiopia prior to the start of the trial in each country, where required and also from the London School of Hygiene & Tropical Medicine Ethics Committee.

### Ancillary studies

Complementary sub-studies are feasible within this large collaborative project and appropriate ethical approval will be sought as necessary.

### Organisation

The DNDi Coordination Team, based mainly at the Coordination Centre, DNDi Africa, Kenya Medical Research Institute, Nairobi are responsible for collation and submission of protocol amendments, organisation of training for trial staff, monitoring and supervision of trial conduct, day to day management of the trial, harvesting data collected at each site, trial monitoring visits and data management, all to Good Clinical Practice (GCP) standards.

### Training

All trial site staff; physicians, nurses, laboratory technicians and pharmacists will receive training on the study protocol, study specific procedures and International Conference on Harmonisation of Technical Requirements for Registration of Pharmaceuticals for Human Use - Good Clinical Practice (ICH-GCP) guidelines [20,21]. Additional training sessions will be provided as required, using external consultants where necessary. Documentation of receipt of training is maintained at the coordination centre.

### Quality control and quality assurance

Suitably qualified Clinical Monitors trained in GCP will regularly visit trial sites to monitor all aspects of the trial including; informed consent procedures, drug accountability, source data verification, adverse event reporting, sample handling, analysis and storage and secure data storage.

### Data collection and data management

Data are to be recorded on 3-part No Carbon Required (NCR) CRFs by site investigators, transcribed from hospital source data. Unique patient identifiers, assigned at the time of randomisation, are linked to unique hospital record numbers. During monitoring visits, CRF data will be cross-checked against hospital source data by clinical monitors. The top sheet for each page of the CRF pages is to be brought to the central Data Centre for double-entry into GCP compliant open-access database software OpenClinica, version 2.7 [22]. Following validation, data will be read into Stata, version 11 special edition [23] and pre-programmed command files will be used to generate lists of data value queries in a thorough data cross-checking process. Query forms are to be automatically generated via Microsoft Access^© ^database software and emailed to trial sites, copied to clinical monitors. The sites will print and make resolutions. At the next monitoring visit, monitors will verify resolutions and deliver to the Data Centre. Data corrections will be programmed in Stata to complete the data cleaning audit trail.

### Publication policy

DNDi, as sponsor, will render all necessary assistance to investigators to ensure publication of results in an international peer-reviewed journal in a timely manner, for the benefit of patients and to inform decision-making with respect to national treatment guidelines for VL. All investigators will be acknowledged when reporting the primary results of the trial through a group authorship. Ancillary studies will acknowledge those involved by name where appropriate.

### Confidentiality

Trial site records will contain names and residential information for each patient to allow follow-up to take place. Only the unique numeric identifier assigned to patients will be extracted from patient records transferred to the central Data Centre. Patient data will be kept securely at trial sites under the responsibility of the site investigator.

### Audit

During the course of the trial, site audits will be undertaken to assess compliance to ICH GCP guidelines. Specific issues to be assessed include adherence to the protocol and standard operating procedures (SOP), consent, laboratory practise, documentation and record keeping. All areas of non-compliance will be addressed by the trial coordination centre.

### Termination of the study

On approaching the end of planned recruitment, the trial coordination office will send written instructions by email to each site to advise on the date to cease recruitment. All patients will be followed up as per the protocol, data collected and cleaned. Once the data lock has been completed, site close out visits will be performed by the coordination team and clinical monitors. A decision for premature termination will be taken in consultation and agreement with the sponsor, investigators and DSMB. All relevant ethics committees and regulatory authorities will also be informed of the reason for termination. Trial master files and CRFs will be archived by the coordination centre in Nairobi for 15 years.

### Indemnity

DNDi as sponsor holds clinical trial insurance to indemnify the study participants for any injury or harm, which occurs during the performance of the trial.

## Discussion

Given the pressing need to develop new treatments for VL in East Africa, this trial aims to meet both phase II and III development objectives by identifying an effective dosage and demonstrating non-inferiority in a suitably powered study. Hence, this trial will allow the evaluation of an effective single-dose regimen and to inform a strategy on use of AmBisome^® ^in the region. The use of a combined phase II and III approach will also allow a cost effective approach considering the hurdles faced in conducting and running a clinical trial in very remote, resource limited settings of VL endemicity. The conduct of the trial through a multi country research platform (LEAP) will also facilitate its implementation, minimise duplication of efforts and allow regional capacity building. The expected outcome will be the identification of an efficacious single dose that can also be used as part of a combination treatment.

## List of Abbreviations

VL: Visceral Leishmaniasis; IM: intramuscular; IV: intravenous; DNDi: Drugs for Neglected Diseases initiative; LEAP: Leishmaniasis East Africa Platform; ITT: Intention-to-Treat; PP: Per Protocol; CTC: common terminology criteria; SSG: Sodium Stibogluconate; SAE: Serious Adverse Event; AE: Adverse Event; DSMB: Data and Safety Monitoring Board; NCR: No Carbon Required; CRF: CaseReport Form; ICH: International Conference on Harmonisation of Technical Requirements for Registration of Pharmaceuticals for Human Use; GCP: Good Clinical Practice; SOP: Standard Operating Procedures.

## Competing interests

DNDi, as sponsor, is funding the trial and costs relating to open-access publication of manuscripts resulting from this research. MW, RO, SE and MB are current employees of DNDi.

## Authors' contributions

All authors have read and approved the final manuscript. Investigators (AH, MW, EAGK, AM, SY), statisticians from LSHTM (TE, PS) and representatives from the sponsor, DNDi, (CR, MB, SE) designed the study. TE and RO drafted this submission.
